# Supplementary material for: Are we missing the forest for the trees? Conspecific negative density dependence in a temperate deciduous forest
Source: PLoS One. 2021 Jul 15;16(7):e0245639. doi: 10.1371/journal.pone.0245639 (PMC8282035; doi:10.1371/journal.pone.0245639)
Supplement: S1 Table — We report any pooled point pattern as overdispersed if it has a significantly positive slope and any pooled point pattern as clustered if it has a significantly negative slope. (DOCX) [file pone.0245639.s001.docx]

**Table S1: Linear estimates of the relationship between L and distance for all pooled point patterns at Powdermill Nature Reserve.** We report any pooled point pattern as overdispersed if it has a significantly positive slope and any pooled point pattern as clustered if it has a significantly negative slope.

| **Point pattern** | **Df** | **T Stat** | **P value** | **Figure** |
| --- | --- | --- | --- | --- |
| All individuals | 2242 | 11.706 | <2 x e-^16^ | 1a |
| All individuals, <0.5 | 792 | 0.07535 | 0.000145 | 1b |
| All individuals , 0.5 - 1m | 1238 | 4.243 | 2.43 x e^-5^ | 1b |
| All individuals, 1-5 m | 1133 | 5.22 | 2.25 x e^-7^ | 1b |
| All individuals, >5 m | 441 | -5.914 | 7.65x e^-9^ | 1b |
| Overstory | 1069 | 8.737 | <2 x e^-16^ | 2a |
| Understory | 916 | 10.191 | <2 x e^-16^ | 2a |
| Overstory, <0.5 | 387 | -5.441 | 9.41 x e^-8^ | 2b |
| Overstory, 0.5-1 | 486 | 4.82 | 1.93 x e^-6^ | 2b |
| Overstory, 5-1 | 471 | 6.14 | 1.75 x e^-9^ | 2b |
| Overstory, >5 | 190 | -3.007 | 0.003 | 2b |
| Understory, <0.5 | 403 | 0.422 | 0.673 | 2c |
| Understory, 0.5-1 | 414 | 0.529 | 0.597 | 2c |
| Understory, 1-5 | 386 | -1.596 | 0.111 | 2c |
| All individuals, bird dispersed | 1171 | 8.594 | <2 x e^-16^ | 3a |
| All individuals, animal dispersed | 202 | 11.97 | <2 x e^-16^ | 3a |
| All individuals, wind dispersed | 814 | 6.973 | 6.43 x e^-12^ | 3a |
| Canopy, bird dispersed | 253 | 5.482 | 1.02 x e^-7^ | 3b |
| Canopy, wind dispersed | 610 | 6.341 | 4.40 x e^-10^ | 3b |
| Understory, bird dispersed | 916 | 8.12 | 1.49 x e^-15^ | 3b |
| Understory, wind dispersed | 202 | 10.897 | <2 x e^-16^ | 3b |
