# Supplementary material for: Are we missing the forest for the trees? Conspecific negative density dependence in a temperate deciduous forest
Source: PLoS One. 2021 Jul 15;16(7):e0245639. doi: 10.1371/journal.pone.0245639 (PMC8282035; doi:10.1371/journal.pone.0245639)
Supplement: S2 Table — To make a more conservative estimate of significance, we calculated the P value for each pooled point pattern using the standard deviation of the L estimates and the number of points contributing to each point pattern. We used the number of points contributing rather than the number of L estimates because L is calculated 51 times (each 10 cm distance bin) for each individual point pattern resulting in an inflated degrees of freedom for the overall model. We then calculated the t statistic as the slope/standard error and used a T table to find the estimated P value for a two-tailed t test. We report the P value for each T statistic at the closest degrees of freedom on the table to our degrees of freedom that was not greater than the actual degrees of freedom (i.e. for a degrees of freedom of 204, we report the p-value for 200 degrees of freedom). This analysis may be overly conservative because the variance, standard deviation, and standard error are calculated based on the L estimates which have a higher variance (as they are calculated 51 times per point) than the average L estimate for each point. (DOCX) [file pone.0245639.s002.docx]

**Table S2: Linear estimates of the relationship between L and distance for all pooled point patterns utilizing degrees of freedom based on the number of points represented by each point pattern rather than the degrees of freedom based on the number of L estimates.** To make a more conservative estimate of significance, we calculated the P value for each pooled point pattern using the standard deviation of the L estimates and the number of points contributing to each point pattern. We used the number of points contributing rather than the number of L estimates because L is calculated 51 times (each 10 cm distance bin) for each individual point pattern resulting in an inflated degrees of freedom for the overall model. We then calculated the t statistic as the slope/standard error and used a T table to find the estimated P value for a two-tailed t test. We report the P value for each T statistic at the closest degrees of freedom on the table to our degrees of freedom that was not greater than the actual degrees of freedom (i.e. for a degrees of freedom of 204, we report the p-value for 200 degrees of freedom). This analysis may be overly conservative because the variance, standard deviation, and standard error are calculated based on the L estimates which have a higher variance (as they are calculated 51 times per point) than the average L estimate for each point.

| **Point pattern** | **N** | **Df** | **Slope** | **Standard Error** | **T Stat** | **P Value** | **Figure** |
| --- | --- | --- | --- | --- | --- | --- | --- |
| All individuals | 704 | 702 | 1.265 | 0.580 | 2.179 | <0.05 | 1a |
| All individuals, <0.5 | 206 | 204 | 0.467 | 0.118 | 3.960 | >0.1 | 1b |
| All individuals , 0.5 - 1m | 181 | 179 | 1.490 | 1.156 | 1.289 | <0.05 | 1b |
| All individuals, 1-5 m | 171 | 169 | 1.039 | 0.636 | 1.635 | <0.05 | 1b |
| All individuals, >5 m | 42 | 40 | -0.415 | 0.320 | -1.296 | <0.4 | 1b |
| Overstory | 338 | 336 | 1.740 | 0.527 | 3.298 | <0.002 | 2a |
| Understory | 352 | 350 | 0.828 | 0.228 | 3.629 | <0.001 | 2a |
| Overstory, <0.5 | 97 | 95 | -0.140 | 0.080 | -1.745 | <.10 | 2b |
| Overstory, 0.5-1 | 96 | 94 | 3.060 | 2.122 | 1.442 | <.10 | 2b |
| Overstory, 5-1 | 89 | 87 | 2.103 | 1.147 | 1.833 | <.10 | 2b |
| Overstory, >5 | 22 | 20 | -0.348 | 0.538 | -0.647 | <0.4 | 2b |
| Understory, <0.5 | 109 | 107 | 0.0322 | 0.210 | 0.154 | <0.5 | 2c |
| Understory, 0.5-1 | 85 | 83 | 0.020 | 0.124 | 0.162 | <0.5 | 2c |
| Understory, 1-5 | 97 | 95 | -0.067 | 0.125 | -0.536 | <0.5 | 2c |
| All individuals, bird dispersed | 312 | 310 | 1.160 | 0.387 | 3.001 | <0.01 | 3a |
| All individuals, animal dispersed | 53 | 51 | 0.760 | 0.192 | 3.953 | <0.01 | 3a |
| All individuals, wind dispersed | 316 | 314 | 1.541 | 0.526 | 2.930 | <0.01 | 3a |
| Canopy, bird dispersed | 50 | 48 | 2.342 | 1.475 | 1.588 | <0.1 | 3b |
| Canopy, wind dispersed | 245 | 243 | 1.822 | 0.675 | 2.698 | <0.01 | 3b |
| Understory, bird dispersed | 262 | 260 | 0.832 | 0.284 | 2.933 | <0.01 | 3b |
| Understory, wind dispersed | 33 | 31 | 0.698 | 0.254 | 2.748 | <0.05 | 3b |
