# Supplementary material for: Are we missing the forest for the trees? Conspecific negative density dependence in a temperate deciduous forest
Source: PLoS One. 2021 Jul 15;16(7):e0245639. doi: 10.1371/journal.pone.0245639 (PMC8282035; doi:10.1371/journal.pone.0245639)
Supplement: S3 Table — We classified species using the Flora of North America species descriptions. If a species had an average height of 5 m or higher, we classified it as a canopy species. If a species had an average height of 5 m or lower, we classified it as an understory species. We based our dispersal syndrome on the description of seed morphology. (DOCX) [file pone.0245639.s003.docx]

**Table S3. List of species from Powdermill Nature Reserve.** We classified species using the Flora of North America species descriptions. If a species had an average height of 5 m or higher, we classified it as a canopy species. If a species had an average height of 5 m or lower, we classified it as an understory species. We based our dispersal syndrome on the description of seed morphology.

| **Species name** | **Common name** | **Growth form** | **Dispersal syndrome** |
| --- | --- | --- | --- |
| *Acer pensylvanicum* | Striped maple | Understory | Wind |
| *Acer rubrum* | Red maple | Canopy | Wind |
| *Acer saccharum* | Sugar maple | Canopy | Wind |
| *Berberis thunbergii* | Japanese barberry | Understory | Bird |
| *Betula lenta* | Black birch | Canopy | Wind |
| *Carpinus caroliniana* | Musclewood | Understory | Wind |
| *Carya glabra* | Mockernut hickory | Canopy | Animal |
| *Carya ovata* | Shagbark hickory | Canopy | Animal |
| *Cornus florida* | Flowering dogwood | Understory | Bird |
| *Crataegus sp.* | Hawthorn | Understory | Bird |
| *Fagus grandifolia* | American beech | Canopy | Wind |
| *Fraxinus americana* | White ash | Canopy | Wind |
| *Hamamelis virginiana* | Witch hazel | Understory | Self |
| *Lindera benzoin* | Spice bush | Understory | Bird |
| *Liriodendron tulipifera* | Tuliptree | Canopy | Wind |
| *Magnolia acuminata* | Cucumber magnolia | Canopy | Bird |
| *Nyssa sylvatica* | Black gum | Canopy | Bird |
| *Prunus serotina* | Black cherry | Canopy | Bird |
| *Prunus virginiana* | Bird cherry | Understory | Bird |
| *Quercus alba* | White oak | Canopy | Animal |
| *Quercus prinus* | Chestnut oak | Canopy | Animal |
| *Quercus rubra* | Red oak | Canopy | Animal |
| *Rosa multiflora* | Multiflora rose | Understory | Bird |
| *Rubus sp.* | Bramble | Understory | Bird |
| *Toxicodendron radicans* | Poison Ivy | Understory | Bird |
| *Vaccinium corymbosum* | Highbush blueberry | Understory | Bird |
| *Viburnum acerifolium* | Maple-leaf viburnum | Understory | Bird |
| *Viburnum prunifolium* | Blackhaw viburnum | Understory | Bird |
